# Research on high sensitivity piezoresistive sensor based on structural design

**DOI:** 10.1186/s11671-024-03971-4

**Published:** 2024-05-16

**Authors:** Wei Li, Xing Liu, Yifan Wang, Lu Peng, Xin Jin, Zhaohui Jiang, Zengge Guo, Jie Chen, Wenyu Wang

**Affiliations:** 1 Lutai School of Textile and Apparel, Shandong University of Technology, Zibo, 255000 People’s Republic of China; 2 Key Laboratory of Clean Dyeing and Finishing Technology of Zhejiang Province, Shaoxing University, Shaoxing, Zhejiang Province People’s Republic of China; 3 School of Textile Science and Engineering, Tiangong University, Tianjin, 300387 People’s Republic of China; 4 State Key Laboratory of Biobased Fiber Manufacturing Technology, China Textile Academy, Beijing, People’s Republic of China; 5 PLA Naval Medical Center, Shang Hai, People’s Republic of China; 6 School of Materials Science and Engineering, Tiangong University, Tianjin, 300387 People’s Republic of China

**Keywords:** Piezoresistive sensor, Pressure sensor, High-sensitivity, Wide detection range, Microstructure, Three-dimensional framework structure, Flexible sensor, Design

## Abstract

With the popularity of smart terminals, wearable electronic devices have shown great market prospects, especially high-sensitivity pressure sensors, which can monitor micro-stimuli and high-precision dynamic external stimuli, and will have an important impact on future functional development. Compressible flexible sensors have attracted wide attention due to their simple sensing mechanism and the advantages of light weight and convenience. Sensors with high sensitivity are very sensitive to pressure and can detect resistance/current changes under pressure, which has been widely studied. On this basis, this review focuses on analyzing the performance impact of device structure design strategies on high sensitivity pressure sensors. The design of structures can be divided into interface microstructures and three-dimensional framework structures. The preparation methods of various structures are introduced in detail, and the current research status and future development challenges are summarized.

**Supplementary Information:**

The online version contains supplementary material available at 10.1186/s11671-024-03971-4.

## Introduction

Tactile perception is one of the main functions of human perception of the external environment. In recent years, flexible sensors that simulate human natural sensing function have attracted much attention in the fields of artificial intelligence [1–3], electronic skin [4–7], software robots [8, 9] and human–computer interactions [10, 11] due to their good compressibility, flexibility and portability, and have been developed rapidly. At present, many reports focus on flexible and high-sensitive sensors using capacitance [12–14], piezoelectric [15–17], frictional [18, 19] and piezoresistive [20–25] sensing mechanisms. Among these types of sensors, the flexible piezoresistive sensor is composed of conductive electrodes and elastic layer, with simple structure. The conductive modes in the elastic layer can be divided into two categories: electrons and ions. During the application of pressure, the contact area and internal network structure will change, which can form more conductive channels and cause changes in resistance/current. The change of external force can be indirectly fed back through the detection of electrical detection system, and the sensing mechanism and signal readout method are simple and convenient, which has attracted wide attention.

As a kind of tactile sensor, flexible piezoresistive sensor should not only ensure the response to slight pressure, but also have good performance of continuous response under high pressure, so as to meet the requirements of simulating human perception as electronic skin. For example, the intracranial pressure and intraocular pressure of the human body are only 1 kPa and 2 kPa respectively, while the typical contractions of the human body are between 13 and 26 kPa [26]. In addition, the pressure of human body to manipulate external objects is often far more than 10 kPa [27]. Therefore, it is necessary and urgent to improve the sensitivity of the sensor and expand the linear detection range.

For piezoresistive sensors, there are generally two ways to express sensitivity:

1 $${{\text{S}}}_{({\text{sensitivity}})}=\frac{\delta \left(\Delta R/{R}_{0}\right)}{\delta P}\approx \frac{\left({R}_{1}-{R}_{0}\right)}{{R}_{1}}/\left({P}_{1}-{P}_{0}\right)=\left(\frac{{I}_{1}-{I}_{0}}{{I}_{0}}\right)/\left({P}_{1}-{P}_{0}\right)$$

2 $${{\text{GF}}}_{(\text{gauge factor})}=\frac{\left({R}_{1}-{R}_{0}\right)}{{R}_{1}}/\varepsilon =\frac{\left({I}_{1}-{I}_{0}\right)}{{I}_{1}}/\varepsilon$$

Formula (1) is the equation of the ratio of relative current/resistance change to applied pressure, where R (or I) is output resistance (or current), P is the applied pressure, R_0_ (or I_0_) is the resistance (or current) of the sensor without pressure and R_1_ (or I_1_) is the resistance (or current) of the sensor with the pressure applied. Another expression of sensitivity is Gauge factor (formula (2)), where ε is the compressive strain. It can be seen that the sensitivity is related to the resistance change and the size of deformation/pressure.

Therefore, it is important to improve the pressure sensitivity and compressibility of the sensor. Although materials with lower Young's modulus can provide excellent compressibility and good flexibility for devices, deformation saturation often occurs at the deformation of materials with lower modulus, which will lead to a smaller compression range of devices. In order to achieve this goal, researchers have done a lot of work and put forward feasible schemes through structural design and optimization.

Flexible piezoresistive sensors are generally composed of conductive material and elastic substrate material. At present, conductive materials commonly used in pressure sensors include electronic materials (metal nanomaterials [28, 29], carbon materials [30, 31], conductive polymers [32–35] and ionic materials (gel materials) [36, 37]. The addition of conductive materials can be divided into internal embedded type and surface coating type (generally applicable to electronic materials).

The way of internal embedding is to mix various conductive materials with elastic polymer substrate materials, so that the conductive materials are evenly dispersed into the polymer matrix. Due to the conductive material would be covered by polymer, only part of it be exposed, so the conductive materials are not easy to fall off, and changes with the deformation of the substrate structure [38, 39]. For ionic conducting materials, their solid polymers usually contain polymer matrix and ionic electrolyte, the former keeps the matrix rigid, and the latter helps the matrix obtain good conductivity [40]. Under the action of external force, the internal free ions can be redistributed to form a double electric layer at the interface to obtain changes in electrical signals. And the surface coating method is more suitable for electronic conductive materials. It is a method to attach conductive materials to the surface of the substrate or the inner surface of porous structure by dip coating, in-situ polymerization/reduction or magnetron sputtering [41–48]. Due to the limitation of material characteristics, they are difficult for the modified conductive materials to significantly enhance the change of electrical signals during compression. In recent years, the sensitivity of pressure sensor has been improved by some fine strategies, most of which focus on the design and optimization of the substrate structure.

**Fig. 1:**
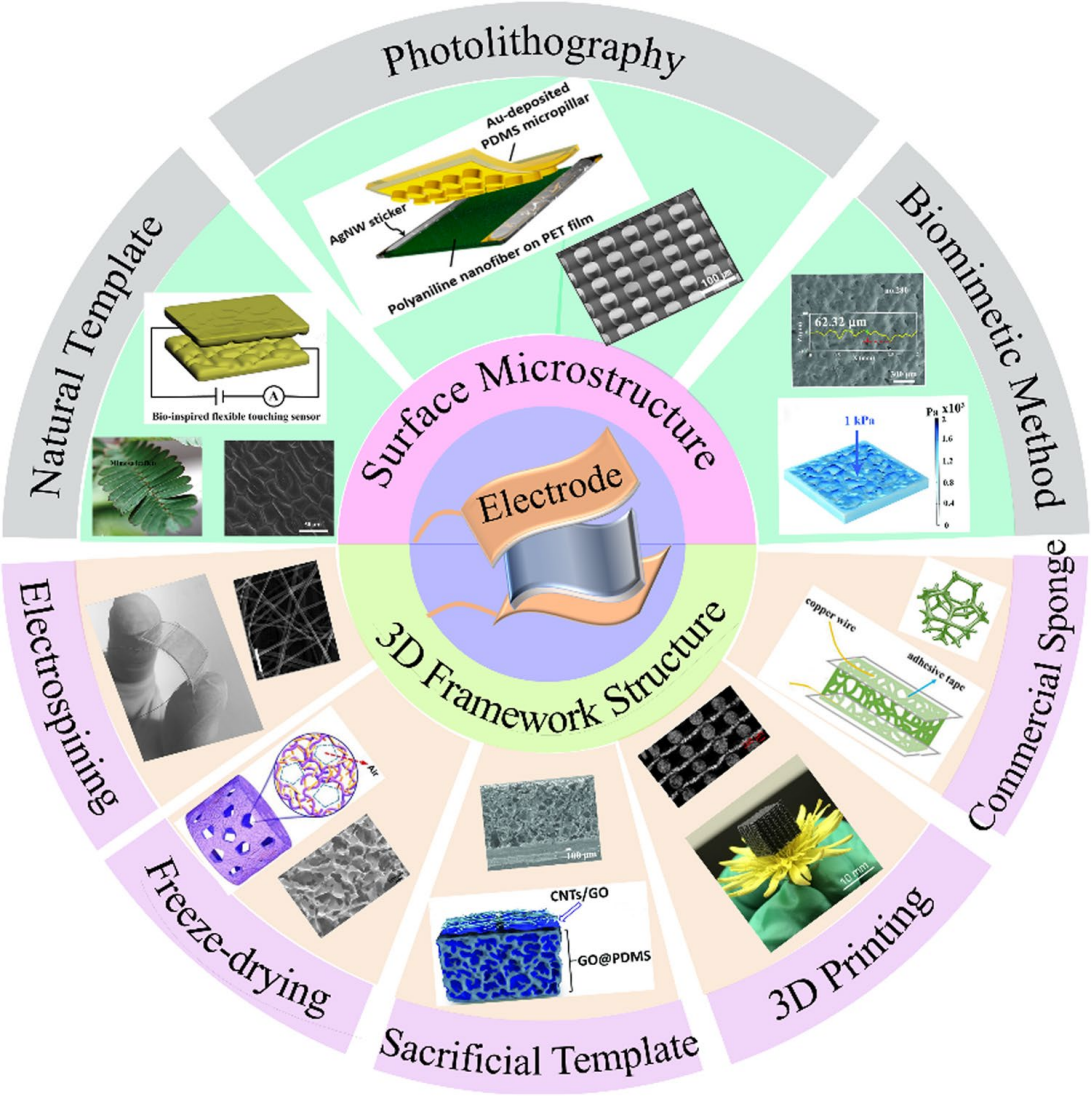
The preparation method of the sensors with high sensitivity. The color of green represents the design methods of surface microstructure. From left to right, Mimosa leaves were selected as templates to prepared the sensor with surface unordered structure. Reproduced with permission [53]. Copyright 2014, John Wiley and Sons. The sensor with micro-column and sharp-pyramid array structure were fabricated by photolithography. Reproduced with permission [54]. Copyright 2015, American Chemical Society. Inspired by human epidermis, an irregular micro-protrusion structure sensor was fabricated by using sandpaper as template to simulate human skin. Reproduced with permission [3]. Copyright 2022, Wiley-VCH GmbH. The color of orange represents the preparation methods of three-dimensional (3D) framework structure. From left to right, transparent and ultra-thin 3D film was prepared by electrospinning. Reproduced with permission [55]. Copyright 2017, Wiley-VCH GmbH. The porous structure was prepared by freeze-drying. Reproduced with permission [56]. Copyright 2020, Elsevier. The porous structure was prepared by using sodium chloride (NaCl) powder as sacrificial template. Reproduced with permission [57]. Copyright 2018, John Wiley and Sons. 3D structure prepared by 3D printing technology. Reproduced with permission [58]. Copyright 2020, American Chemical Society. And commercial PU sponge. Reproduced with permission [59]. Copyright 2018, RSC Pub

According to different device structure designs, substrate materials can be divided into two categories: interface microstructure and three-dimensional (3D) framework structure, as shown in Fig. 1. On the one hand, arterial monitoring, acoustic vibration, breathing frequency and weight monitoring of flowers and feathers are small under pressure. Flexible pressure sensors with small deformation and ultra-high sensitivity have better sensing performance. Flexible pressure sensors based on surface microstructure has good performance. Compared with unstructured elastic films of similar thickness, the microstructures can greatly improve the deformation under the same pressure (or reduce the pressure under the same deformation), thereby increasing the resistance change and obtaining high sensitivity sensors. It can also be adjusted by using different microstructures (size, density and shape) and applied in different fields, as shown in the upper half circle of Fig. 1 [49–51]. On the other hand, in the aspect of human motion monitoring, such as finger and knee joint movement, foot muscle movement and multi-module monitoring of foot sole are used to collect different walking gaits. Because of the great effect of force, it is necessary for the sensor to work under large deformation conditions. 3D framework structure pressure sensors with good porosity are excellent representative of large deformation, as shown in the lower half circle of Fig. 1 [27]. With the increase of pressure, the porous structure is gradually compressed and closed, rather than closed together, which makes the contact area of the sensor gradually increase, thus expanding the pressure detection range [52].

This article provides an overview of the structural design of sensors, which can be divided into interface microstructure and three-dimensional framework structure from different perspectives. The paper comprehensively summarizes the different methods of sensor structural design and explores the impact of structural design on sensor sensitivity. In addition, a detailed analysis was conducted on the interesting post-processing optimization process of the structure. Summarized the different application fields corresponding to different structural designs, as well as future development directions and challenges.

## Method and improvement of high sensitivity sensor based on interface microstructure

Human beings could perceive weight loads as low as 0.1 g·mm^−2^ (about 1 kPa). Although the development of pressure-sensitive artificial skin is exciting, researchers are still trying to advance and obtain the ability to detect lower pressure to meet special requirements of applications. And these tasks are still challenging. Therefore, researchers have proposed a variety of interesting and different preparation methods to obtain high sensitivity sensors through surface microstructure design and improvement [60–62]. These preparation methods will be discussed in detail.

### Preparation methods of interface structure

Different micro/nano structure design of device interface can be used for micro-pressure detection. On the one hand, the bulges of the microstructure can concentrate the stress, and under the action of external force, it can rapidly deform and improve the contact area. On the other hand, the design of the structure can form gaps between the substrate matrix and electrodes, obtain good compressibility, improve the sensitivity and expand the detection range of the sensor [3, 63, 64]. The fabrication of interface microstructure pressure sensor can be done by the following methods: (1) Template method; (2) Bioinspired method. Its substrate material not only includes various polymer materials (PDMS, TPU etc.), but also can be gel films composed of mixed liquid of gel and ionic electrolyte.

#### Template method

There are many kinds of templates for fabricating surface microstructures. Various shapes of micro arrays can be fabricated on silicon substrates by photolithography, or directly use the plant leaves with microstructure surface as the natural template.

**Fig. 2:**
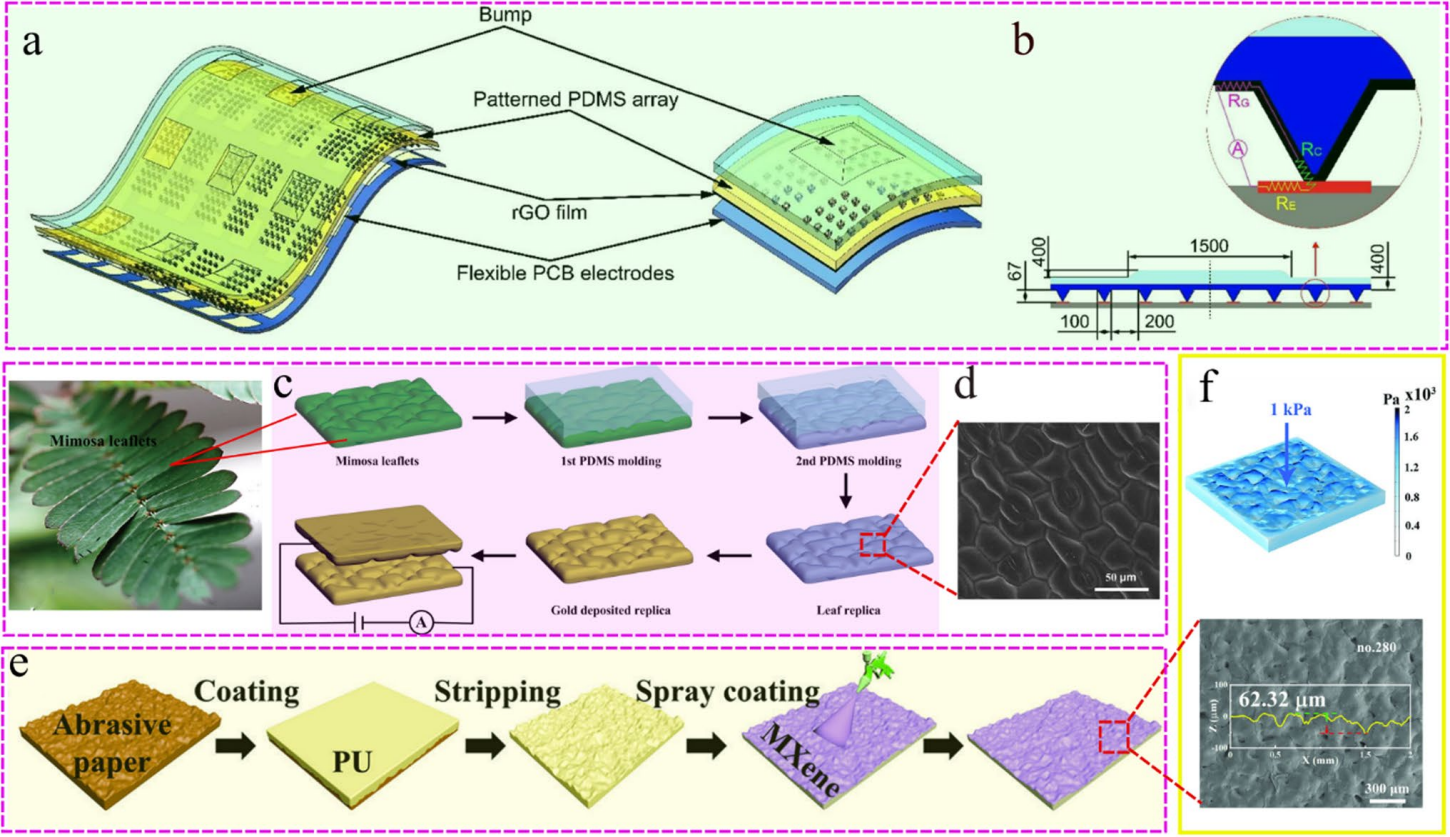
**a** Schematic description of our flexible three-axis tactile sensor architecture. Reproduced with permission [65]. Copyright 2018, ROYAL SOC CHEMISTRY. **b** Cross-section scheme of the sensor structure with parameters. Reproduced with permission [65]. Copyright 2018, ROYAL SOC CHEMISTRY. **c** The schematic diagram of preparing flexible microstructure conductive film with Mimosa leaf as template. Mimosa leaves are washed with deionized water. PDMS mixed solution was poured on the surface of plant leaves, heated in the oven at 60 °C for 4 h to solidify. After obtaining the negative template, the positive template could be obtained by the same method. Finally, Ti/Au electroactive layer was deposited. Reproduced with permission [53]. Copyright 2014, John Wiley and Sons. **d** SEM image (top view) of a PDMS membrane using Mimosa leaf as template. Reproduced with permission [53]. Copyright 2014, John Wiley and Sons. **e** Preparation of spinosum structure flexible substrate coated with MXene. Sandpaper is used as a template. The flexible substrate with random distribution spinosum structure can be obtained by casting PU on the microstructure surface of a sandpaper. After drying, MXene was evenly distributed on the spinosum structure surface of the PU substrate, forming a spinosum MXene/PU layer. Reproduced with permission [3]. Copyright 2022, Wiley-VCH GmbH. **f** Under the pressure of 1 kPa, the simulation results of pressure distribution in the sensitive layer (upper) and the SEM image of MXene/PU layer with randomly distributed spinosum microstructure (lower). Reproduced with permission [3]. Copyright 2022, Wiley-VCH GmbH

*Photolithography technology*. Template method usually uses photolithography or silicon etching processing technology, and designs different patterns on silicon materials as templates [51, 66]. In order to release easily, a layer of release agent (octadecyl trichlorosilane, trichloro-(1H,1H,2H,2H-perfluorooctyl) silane) is usually coated in advance. Then, the mixed solution of elastic material or gel and ionic electrolyte are cast on the template to form films, and the elastomer with surface microstructure is obtained. At present, the commonly used patterning templates include sharp micro-pyramid, micro-column or hemispherical structure [67–69]. By adjusting the parameters of the templates, patterned elastomers with different sizes and densities could be obtained. As shown in Fig. 2a, b, Zhang et al. [65] prepared concave pyramid pattern templates using standard photolithography techniques. A convex micro-pyramid array was formed by transferring polydimethylsiloxane (PDMS) as the substrate material, and a rGO/PDMS flexible sensor was prepared using reduced graphene oxide (rGO) as the conductive layer. Under the pressure applied, the deformation of the pyramid structure will change the contact area between the rGO film and the electrode, enabling the sensor to achieve high sensitivity (1.71 kPa^−1^) at the low-pressure range (0–225 Pa). This method is also applicable to other elastomer materials (PDMS/PU) [70, 71].

More noteworthy is that by selecting different parameters of the micro-pyramid structures, the sensitivity could be adjusted according to different sensing purposes. The different properties of microstructural thin films with different feature sizes lies in the difference between the number of pyramid patterns and the deformation degree of each feature under the same pressure loading. For the same region, microstructure films with smaller feature sizes would include a larger number of pyramidal patterns that would deform with pressure, resulting in a more significant response. In addition, under the same applied pressure, the smaller pyramid structure would deform more seriously and cause greater changes in contact area, so it has better sensitivity to pressure changes [65]. Similarly, the microcolumn structure and hemispherical structure prepared by template method have similar deformation mechanism with micro pyramid structure.

Photolithography technology is a common method for preparing interface microstructures, which endows elastic materials with good tactile sensitivity. However, this technology is a time-consuming and costly process that is not suitable for large-scale expansion and application.

*Natural template method*. Many plants in nature have special survival or self-protection functions. For example, Mimosa will produce stress reaction after being forced to close the leaves, and the excellent "hydrophobic effect" on the surface of lotus leaves is easy to capture air bags. Research has found that many plant leaves and petal surfaces have various shapes of microstructures, which provide ideas for the preparation of interface microstructures [53, 63, 72].

Inspired by this, plant leaves or petals can be directly used as microstructural templates. The leaves of mimosa are composed of many disordered structures, with an average height of about 16 μm and a diameter of about 18.4 μm; the rose petals exhibit a uniform papillary microstructure, with an average diameter of 23 μm and a height of 40 μm; the surface of rose leaves has dense island like microstructures, with an average height of about 10 μm and the surface of acacia leaves is needle like microstructure, with a diameter of about 25 μm and a height of about 300 μm [63, 73]. After cleaning these leaves and petals, the elastic matrix is directly laid on the plant template to obtain negative films with rough structures, and then transferred again to obtain positive films with prominent structures. By combining with conductive materials through deposition, polymerization, and other methods, a high-performance sensing layer can be successfully obtained [60, 74]. As shown in Fig. 2c, d, Su et al. [53] used mimosa leaves as templates to fabricate micro patterned polydimethylsiloxane substrates, and deposited a conductive gold layer on the surface to transport electrons to prepare a flexible sensor with microstructures. This method can also be applied to other plant templates with surface microstructure [63, 72, 73].

These natural structures can effectively avoid complex process designs, reduce costs, and shorten time consumption. However, the size of the template is limited by the area of plant leaves or petals, making it impossible to obtain larger devices. At the same time, the maturity of plant leaves can also affect the formation of natural structures, posing certain challenges to the repetitive preparation of sensors.

#### Bioinspired method

The rapid development of science and technology has brought many convenient services to humanity in various aspects, greatly liberating human hands. However, in many aspects, the biological functions of humans and animals are still superior to some artificial machines, and their unique biological structure and good function also provide direction for further efforts by researchers. For example, plants and animals have their own pressure sensing systems, which help to sense the external environment and avoid crises. As the most important pressure sensing tissue, human skin provides new inspiration and strategy for bionic structure [29, 63]. Although the skin structure cannot be directly obtained as a template, a series of simulation experiments can be carried out to achieve similar functions. Yang et al. [3] prepared a pressure sensor with random distribution spinosum microstructure by imitating human epidermal tissue, as shown in Fig. 2f. Sandpaper is used as a bionic template. The flexible substrate with randomly distributed spiny structure can be obtained by casting polyurethane on the surface of sandpaper microstructure. Interestingly, the stress of this spinosum structures was concentrated on the initial contact peak and can be transferred to the bottom of adjacent peaks, which could increase the change of contact area under pressure (Fig. 2e). In addition, due to the different heights of spinosum structure, their random distribution contributes to a larger linear range [41, 61]. This method provides a good idea for the preparation of interface microstructures, but suitable biomimetic materials and methods require extensive research by researchers.

### Improvement based on surface structure

The design of surface microstructure is based on stress concentration. Although the design of interface microstructures can bring about significant changes in the contact area between the conductive layer and the electrodes under small pressure, such as micro pyramids, micro hemispheres, and irregular structures, making the sensor highly sensitive and responsive. However, with the increase of pressure, the deformation saturation of the pre-contact stress area is no longer sensitive, which would lead to a sharp decrease in sensitivity subsequently, limiting the linear sensing range of the sensor. In order to maintain the high sensitivity and improve the detection range, the structure improvement was proposed based on the surface microstructure.

#### Hierarchical structure of surface

Designing smaller structures based on surface microstructure and forming layering is an effective method to further improve sensitivity.

**Fig. 3:**
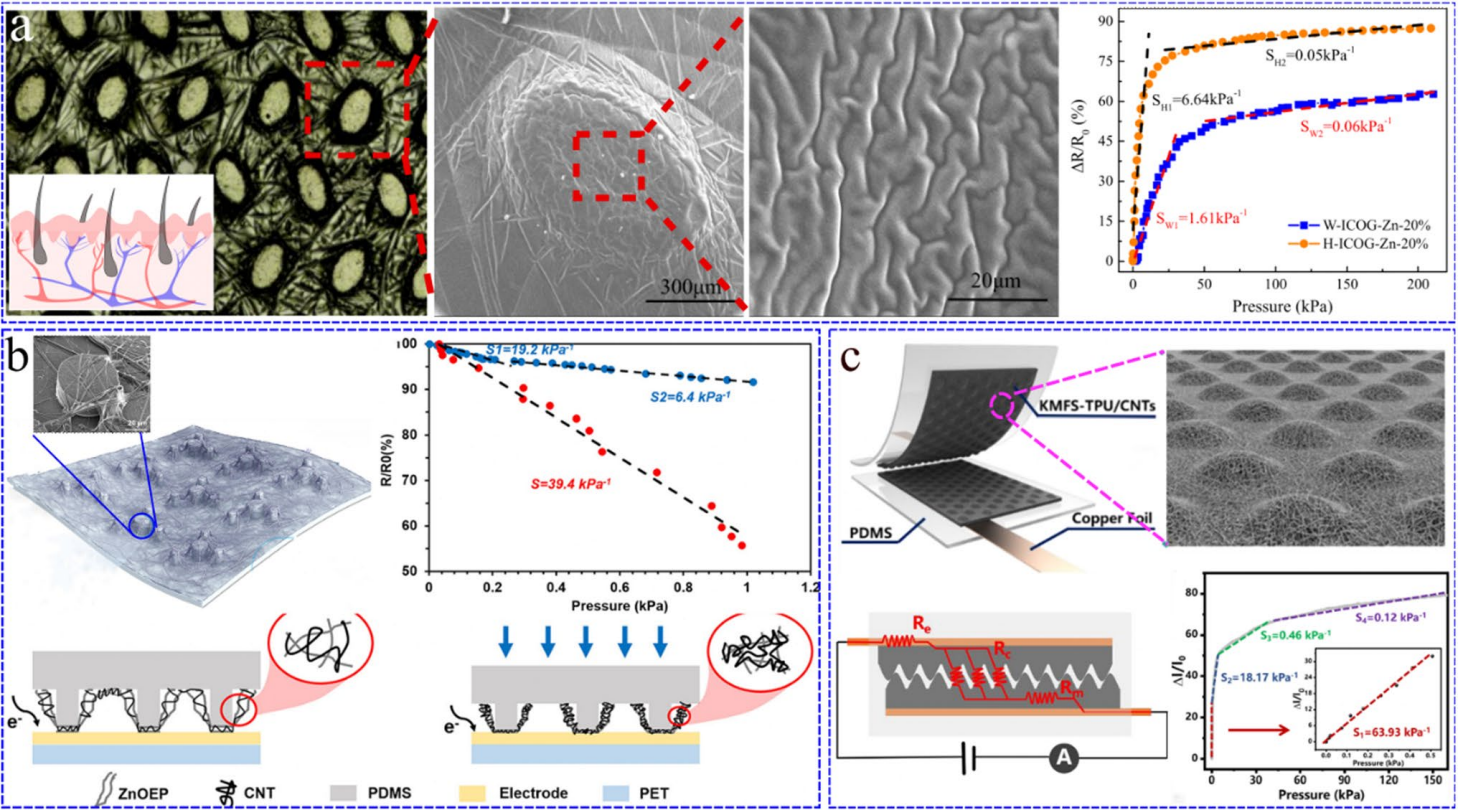
**a** Optical microscope image of gel film with interface hierarchical structure, the illustration of which is the schematic diagram of human skin. On the right side are the local magnified SEM images of gel, which are respectively elliptical bulge microstructures and irregular wrinkle structure. The far right shows the ΔR/R_0_ (%) versus pressure curve of sensors fabricated with wrinkled structure and hierarchical structure over a pressure range of 0–209 kPa. Obviously, the sensor with hierarchical structure has more sensitive pressure response. Reproduced with permission [75]. Copyright 2022, Elsevier. **b** The schematic diagram of cobweb-like structure coated on microcolumn array. The illustration is SEM image of the ZnOEP/CNT network (upper left). Influence of structure design (with/without microcolumn) on sensitivity of pressure sensor under small pressure (0–1 kPa) (upper right). Schematic diagram of working mechanism (without/with pressure applied) of piezoresistive sensor (lower). Reproduced with permission [77]. Copyright 2020, American Chemical Society. **c** Schematic diagram of interlocking structure pressure sensor and magnified SEM image of knoll-like microstructures and fiber-network structures/CNTs (KMFS-TPU/CNTs) membrane (upper); Equivalent circuit analysis diagram of the sensor under applied pressure (lower left); Sensitivity analysis of sensors under wide pressure (0 to 160 kPa). In different pressure ranges, the sensitivities are 63.93 kPa^−1^ (0–0.5 kPa), 18.17 kPa^−1^ (0.5–4 kPa), 0.46 kPa^−1^ (4–30 kPa) and 0.12 kPa^−1^ (30–160 kPa) (lower right). Reproduced with permission [64]. Copyright 2022, Elsevier

In the previous work, our team prepared a conductive gel with high flexibility, high adhesion and recyclability using polyacrylonitrile as the material [75]. In the process of gel film formation, due to the limitation of biaxial force, the gel film forms isotropic buckling structure, similar to the wrinkle structure of human skin. On this basis, the cilia tissue was simulated, and the non-woven fabric with groove structure was used as the flexible template to successfully prepare the gel interface hierarchical structure, as shown in Fig. 3a. For hierarchical structure, with the increase of pressure, the contact protrusions deform separately developed by Archard model [76]. The increase of the number of deformable protrusions and the contact area makes the relationship between the pressure and the contact area almost linear, which makes the sensor obtain high sensitivity response in a wide pressure range (S = 6.64 kPa^−1^, < 11.1 kPa). In addition, inspired by the cobweb, Zhang et al. [77] creatively suspended CNT/zine octaethylphorphyrin (ZnOEP) cobweb-like Nanonetworks on the microcolumn array to obtain the loose multilayer structure. This loose structure has a larger deformation space before they are completely attached to the substrate under the pressure, which leads to higher sensitivity (S = 39.4 kPa^−1^, 0–1 kPa), as shown in Fig. 3b.

#### Interlocking structure

In addition, the design of interlocking structure can also effectively improve the problem that the high sensitivity is limited to the low pressure. Due to the increased contact area and the expansion of deformation range between stacked layers, this double-layer or multi-layer interlocking structure can not only accurately detect the pressure changes during small deformation (such as airflow, breathing and pulse pressure), but also maintain its high sensitivity and linear response in a relatively wide range of pressure [52, 78, 79]. For example, the interlocked of hemispherical structure designed by Wang et al. [43] could be divided into "sphere to sphere" mode and "sphere to groove" mode. The former has large compressible space, and the latter can obtain larger contact area. This structure could make the sensor still produce obvious signal response under high pressure (S = 196 kPa^−1^, 0–10 kPa). In addition, the hierarchical microstructure designed by Yang et al. [64] placed the two layers with microstructure face-to-face through the interlocking design. As shown in Fig. 3c, the effective combination of surface knoll-like microstructures and fiber-network structures enables the sensor to achieve ultra-low detection limits (0.7 Pa), high sensitivity (63.93 kPa^−1^), and relatively wide sensing range under pressure (0–160 kPa).

Table 1 summarizes the initial sensitivity and linear detection range of sensors based on interface structure design. It can be seen that the design of interface microstructure is an effective method to achieve high sensitivity under low pressure. Under the stimulation of small pressure signals, it can accurately capture signal fluctuations and achieve high sensitivity response. And the optimization of interface structure can further improve the linear detection range of the device, effectively expanding its application field under certain conditions. However, sensors designed based on interface structures often have certain limitations in their working range, and there is a high risk of failure and irreversible deformation when subjected to excessive pressure. At the same time, large-scale preparation and high sensitivity detection are very complex and difficult [80].

**Table 1:** Summary of pressure sensor parameters based on different interface structures

Structure design	Sensitivity (KPa^−1^)	Linear sensing range (Pa)	Response time (ms)	References
Micro-pyramid	15.6	60	200	[51]
Micro-pyramid	1907.2	100	0.05	[70]
Hierarchical papillae	1.2	25 K	–	[72]
Elliptical bulges + wrinkle	6.64	11.1 K	–	[75]
Interlocking nano-cones	268.36	200	48	[52]
Interlocking meso-domes	6.258	40	20	[78]
Multilayer interlocking micro-dome	47.7	353 K	20	[79]

## Method and improvement of high sensitivity sensor based on 3D framework structure

In daily life, the force involved in many human activities and work is far greater than 10 kPa. Therefore, the development of flexible electronic skin cannot be limited to the pursuit of high sensitivity, and its linear detection range is also important. Although the improved method of surface microstructure can improve the linear range of sensitivity, there are still limitations. On this basis, researchers further proposed a three-dimensional framework structure to improve the relationship between sensitivity and detection range.

### Preparation methods of 3D framework structure

3D framework structure is a kind of porous elastic material, with high porosity and certain elastic deformation, which is conducive to the occurrence of strain and resistance changes under pressure. In the past few years, 3D framework structure has been widely studied. According to the preparation method, it can be divided into the following categories: (1) electrospinning technology; (2) Freeze drying technology; (3) Sacrificial template method; (4) 3D printing technology and (5) commercial materials.

#### Electrospinning technology

Electrospinning is an excellent technology for producing nanofiber films with large specific surface area, high aspect ratio and 3D structure [81]. Through the electrostatic repulsion force between the charged surfaces, the viscous fluid is continuously released to form fibers. The fibers are collected on a fixed material and a collector to form a 3D nanofiber film [82]. Moreover, the diameter of the fiber can be adjusted according to changes in process parameters such as the distance between the needle and the collecting device, the diameter of the needle and the voltage [83, 84].

**Fig. 4:**
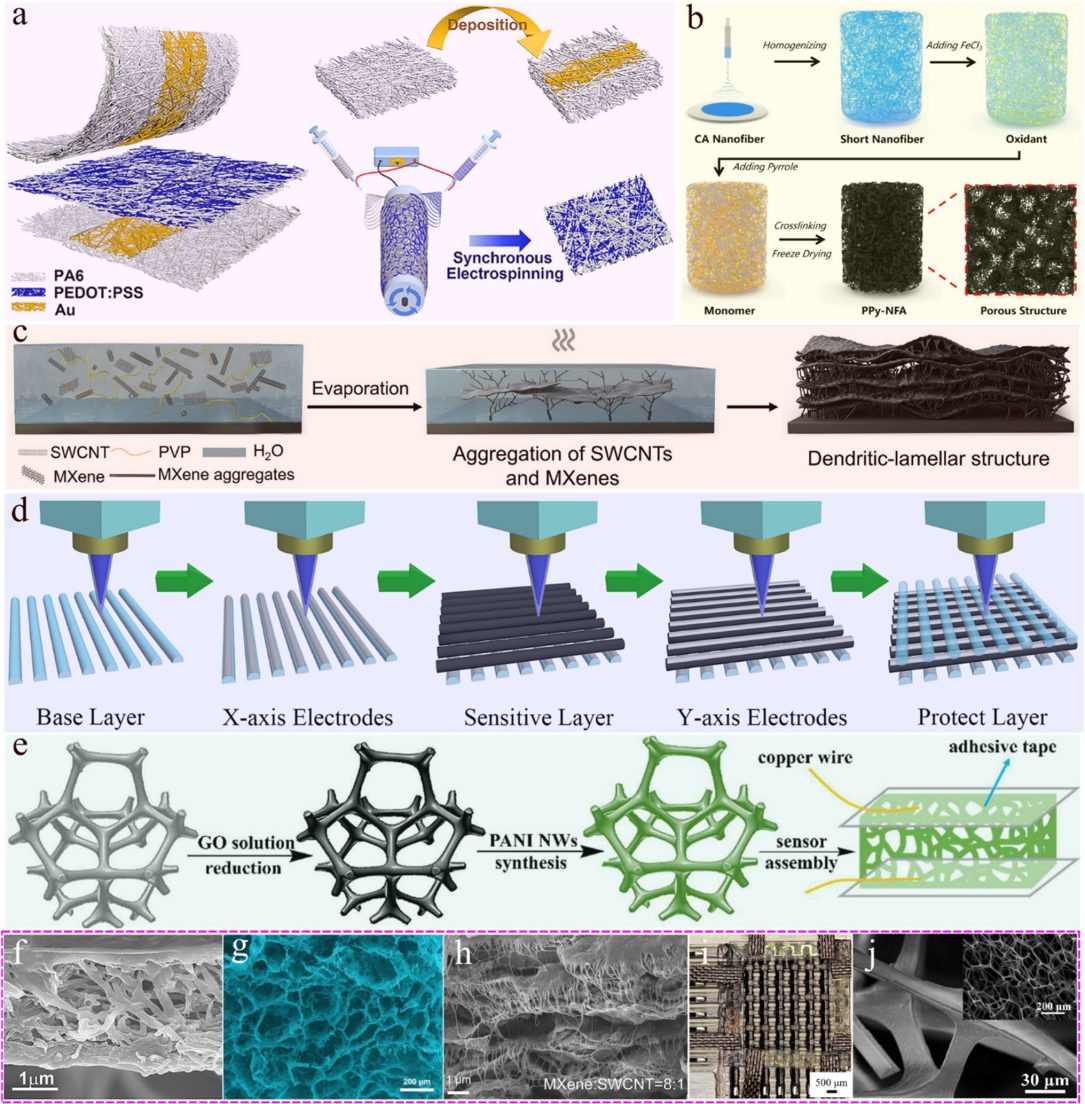
**a** The process flow of preparing poly(3,4-ethylenedioxythiophene):poly(styrenesulfonate)/polyamide 6 (PEDOT:PSS/PA6) nanofiber membranes pressure sensor by electrospinning technology. Schematic diagram of the interlayer structure of a PEDOT:PSS/PA6 nanofiber pressure sensor coated with gold electrodes (left). The preparation process of gold electrode and the process flowchart of PEDOT:PSS/PA6 nanofiber (right). Reproduced with permission [87]. Copyright 2022, American Chemical Society. **b** Schematic diagram of preparing all nanofiber based polypyrrole crosslinked nanofiber aerogel (PPy-NFA) using freeze-drying method. Firstly, cellulose acetate (CA) nanofibers were prepared through electrospinning technology and crushed to obtain short nanofibers as skeleton materials. Then, conductive PPy-NFA with well-developed pores was obtained through in-situ polymerization and freeze-drying technology. Reproduced with permission [90]. Copyright 2022, Elsevier. **c** Schematic diagram of the self-assembly process of dendritic MXene/MWCNT/PVP film induced by water-evaporation. MXene/MWCNT/PVP ink was dip-coated on the surface of natural rubber for water evaporation in the experiment. The SWCNTs self-assembled with MXene in lamellas and further connect with each other through SWCNT bundles, forming a porous dendritic structure. Reproduced with permission [94]. Copyright 2020, Elsevier. **d** Process diagram for preparing E-skin using 3D printing technology. By synthesizing flexible pressure sensitive ink and substrate ink, a layered wooden pile structure was formed using 3D printing technology to achieve a high-density tactile sensing array. Reproduced with permission [104]. Copyright 2022, Copyright 2022, American Chemical Society. **e** Process diagram for preparing flexible pressure sensors based on commercial sponges. Commercial sponges obtain conductivity through coating reduced graphene oxide (rGO) and in-situ synthesis of polyaniline nanowires (PANI NWs), and can be directly assembled with electrodes to form sensors for use. Reproduced with permission [59]. Copyright 2018, RSC Pub. **f** Cross-sectional SEM image of PEDOT:PSS/PA6 fibers. Reproduced with permission [87]. Copyright 2022, American Chemical Society. **g** SEM image of the porous structure of PPy-NFA. Reproduced with permission [90]. Copyright 2022, Elsevier. **h** Cross-sectional SEM images of the MXene/MWCNT/PVP film with a mass ratio of 8:1:2 for MXene/MWCNT/PVP. Reproduced with permission [94]. Copyright 2020, Elsevier. **i** optical image of E-skin prepared through 3D printing technology. Reproduced with permission [104]. Copyright 2022, American Chemical Society. **j** SEM image of melamine sponge. Reproduced with permission [59]. Copyright 2018, RSC Pub. Figure **f**–**j** are the SEM images of the 3D structure prepared by the five different methods in figure **a**–**e**

Nanofiber films obtained by electrospinning have been widely studied due to their ultra-light weight, large specific surface area and good flexibility [23, 85, 86]. As shown in Fig. 4a, Zhou et al. [87] prepared an ultra-thin multilayer nanofiber network composed of poly (3,4-ethylenedioxythiophene):poly (styrenesulfonate) and polyamide 6 (PEDOT:PSS/PA6) through electrospinning technology, and constructed a flexible piezoresistive pressure sensor. And the cross-sectional SEM image of PEDOT:PSS/PA6 fibers is shown in Fig. 4f. The interweaving of fibers provides rich contact points for the loading process and successfully achieves ultrahigh sensitive response. This simple technology provides the possibility for large-scale mass production of wearable sensing devices. At the same time, due to the lightweight nature of electrospinning films, they tend to become saturated under relatively small pressure, which to some extent limits the expansion of the detection range.

#### Freeze drying

Freeze drying is a dehydration technology, also known as sublimation drying. It is a method to prepare porous structure by freezing the water or solvent in the material below the freezing point and then sublimating it from the solid state to the gaseous state in a higher vacuum. In the process of freeze-drying, the pore size and pore distribution can be adjusted by controlling the experimental parameters (such as freezing rate, drying speed, etc.) [88, 89]. Its abundant pore structure and excellent mechanical properties make it an ideal carrier for constructing highly sensitive flexible piezoresistive sensors. In combination with electrospinning technology, Qin et al. [90] prepared cellulose acetate (CA) nanofibers as a skeleton structure, and then successfully constructed three-dimensional polypyrrole (PPy)/CA nanofiber aerogel through in-situ polymerization and freeze-drying technology. The preparation process and SEM image of PPy-NFA porous structure are shown in Fig. 4b and g, respectively. Thanks to the unique microstructure of nanofiber aerogel, the prepared flexible piezoresistive sensor has ultra-high sensitivity (60.28 kPa^−1^) and a wide pressure range (0–24 kPa). This method has simple process, excellent performance, and good application prospects in the wearable field. However, not all materials can adopt this method, as it may bring some adverse factors to the sensor, such as brittleness. Moreover, this method often uses high-temperature carbonization to construct three-dimensional conductive networks, which also reduces strength and compressibility, and increases preparation costs [91].

#### Method of sacrificial template

Sacrificial template method is an effective method for preparing three-dimensional porous structures with adjustable pore size. This method involves adding a certain of sacrificial materials to the substrate material as topological impurities for deployment, which can serve as a temporary framework to assist in the formation of three-dimensional structures. Due to occupying a certain volume, it can be removed by methods such as decomposition, dissolution, melting, and etching to obtain a three-dimensional porous structure [92–95]. Currently, the commonly used templates for flexible sensors include sodium chloride powder (NaCl), sugar, poly (ethylene glycol), citric acid monohydrate (CAM), etc. [27, 95–97]. Huang et al. [80] used copper nanowires as sacrificial templates and successfully removed copper using the acid generated during pyrrole polymerization, forming a good necklace like structure. It shows better sensing performance than traditional spherical aerogel, with high sensitivity (S_min_ > 1.1 kPa^−1^) and ultrabroad sensing range (0.12–400 kPa). It is worth noting that, the as-designed porous aerogel and polyacrylamide (PAAm) hydrogel were combined to effectively overcome the sensor saturation under relatively small pressure. Its sensing pressure ranges from weak pulse waves in human physiological signals to pressure applied by vehicles, with a wide detection range (0.12–400 kPa). In addition, Ni foam can also be used as a three-dimensional framework material, and the Ni skeleton can be fully etched with hydrochloric acid solution after the surface coating material to obtain a three-dimensional structure with multi-level pore [98]. Zhao et al. [93] used CAM as a sacrificial template to control the porosity of the sponge structure by adjusting the volume ratio of CAM to PDMS (elastic substrate), and creatively developed a three-layer porous PDMS/AgNP sponge structure with different porosity. The sponge with a porosity of 45%, 60%, and 75% from top to bottom in its three layers reduces the Young's modulus. Under the application of pressure, the deformation from the third layer to the first layer is significantly increased, and the detection range can reach 200 kPa. In addition, Chen et al. [94] prepared a porous layered dendritic self-assembly structure based on evaporation of water from the mixed aqueous solution of single-walled carbon nanotubes (SWCNTs) and polyvinylpyrrolidone (PVP). The preparation process and the SEM image of the 3D structure are shown in Fig. 4c and h, respectively. Compared with the brittleness of the structure prepared by freeze-drying method, it has higher flexibility and stronger adhesion.

The sacrificial template method can control the change of porosity by adjusting the content of the template, which could change the performance of the sensor. However, the template content is not easily too high, which may lead to problems such as difficulty in forming mixed materials (elastic substrates and template materials) and easy collapse, which can lead to a certain limit in porosity.

#### 3D printing technology

3D printing is a kind of low-cost and rapid prototyping technology, which is based on digital model file, through layer-by-layer printing to construct object structure. By developing ink suitable for flexible sensors and meeting the requirements of typical rheology, 3D structure could be obtained by printing to the substrate of the required 3D model [39, 58, 99–103]. Sang et al. [104] successfully prepared electronic skin with high resolution and breathability by synthesizing flexible sensitive ink and substrate ink, using 3D printing technology to form a clear 3D woodpile-shape and maintaining good contact with adjacent layers. The results are shown in Fig. 4d and i. At the same time, the experiment further added NaCl as a sacrificial template to the ink, obtaining an internal porous structure. The synergistic effect of multiple structures not only achieved high sensitivity response of the pressure sensor, but also achieved penetration and growth of human hair with the high porosity retained by its 3D printing technology, fully considering the wearability of electronic skin.

3D printing technology could control the piezoresistive coefficient of the sensing layer by changing the weight ratio of conductive material and insulating material, and adjust the digital model to control the structure. But this method needs the support of digital printing technology.

#### Commercial materials

One way to get 3D structure simply and quickly is to buy commercial sponge directly. At present, there are many sponge products with different materials and functions on the market, including natural sponge and industrial sponge [44, 105–107]. Sponge selection based on flexible sensor must have good flexibility, resilience and biocompatibility under stress and strain. Polyurethane (PU) or melamine sponge has been widely studied because of its soft handle, high opening structure and compressibility. The flowchart of preparing a flexible pressure sensor using melamine sponge as the substrate is shown in Fig. 4e and SEM image is shown in Fig. 4j [59]. The selected sponge can be directly cleaned with ethanol and deionized water, and combined with different conductive materials (CNT, GO, Ag NPs, etc.) through impregnation, grafting, vapor deposition, hydrothermal method, adhesive, coating, and plasma treatment, etc. to form a conductive sensing layer [28, 44, 45, 47, 108–114]. The preparation of sensors based on commercial sponges is fast and easy to operate, but their pore size and porosity cannot be changed arbitrarily, and the conductive layer coated on the outside may have detachment phenomenon.

Flexible sensors with 3D framework structure generally have a wide detection range and relatively high sensitivity, which are mainly used in wearable electronic devices and human motion monitoring. This is based on the high porosity and good flexibility of 3D framework structure, which can withstand a wide range of pressure and produce large deformation, and ensure relatively high sensitivity response under large deformation conditions.

### Improvement based on 3D framework structure

The design mechanism of 3D framework structure is based on the presence of pores, which make them more prone to deformation under the pressure applied. As the applied pressure increases, the contact area changes constantly, which improve the sensitivity of sensors. However, due to the relatively large pore structure brought about by the framework architecture, the electrical signal changes are relatively small under small pressure. For example, Liu et al. [115] deposited highly conductive graphene and flame-retardant montmorillonite alternately on a three-dimensional melamine sponge to prepared a flame-retardant flexible sensor. Within a strain of 20%, its gauge factor value was only 0.26. Continuing to increase the strain to 60%, gauge factor value rapidly increases to 0.77. When the initial pressure is small, the sponge skeleton bends, and its electrical signal changes were mainly determined by the tunnel effect between the adjacent conductive nano-materials. As the pressure increases, the bending of the sponge skeleton increases, causing the skeleton to contact and compress each other, gradually densified the conductive network, and leading to an increase in conductivity. Therefore, researchers have attempted various methods to improve sensitivity performance in low pressure ranges based on three-dimensional frame structures.

#### Fracture effect

**Fig. 5:**
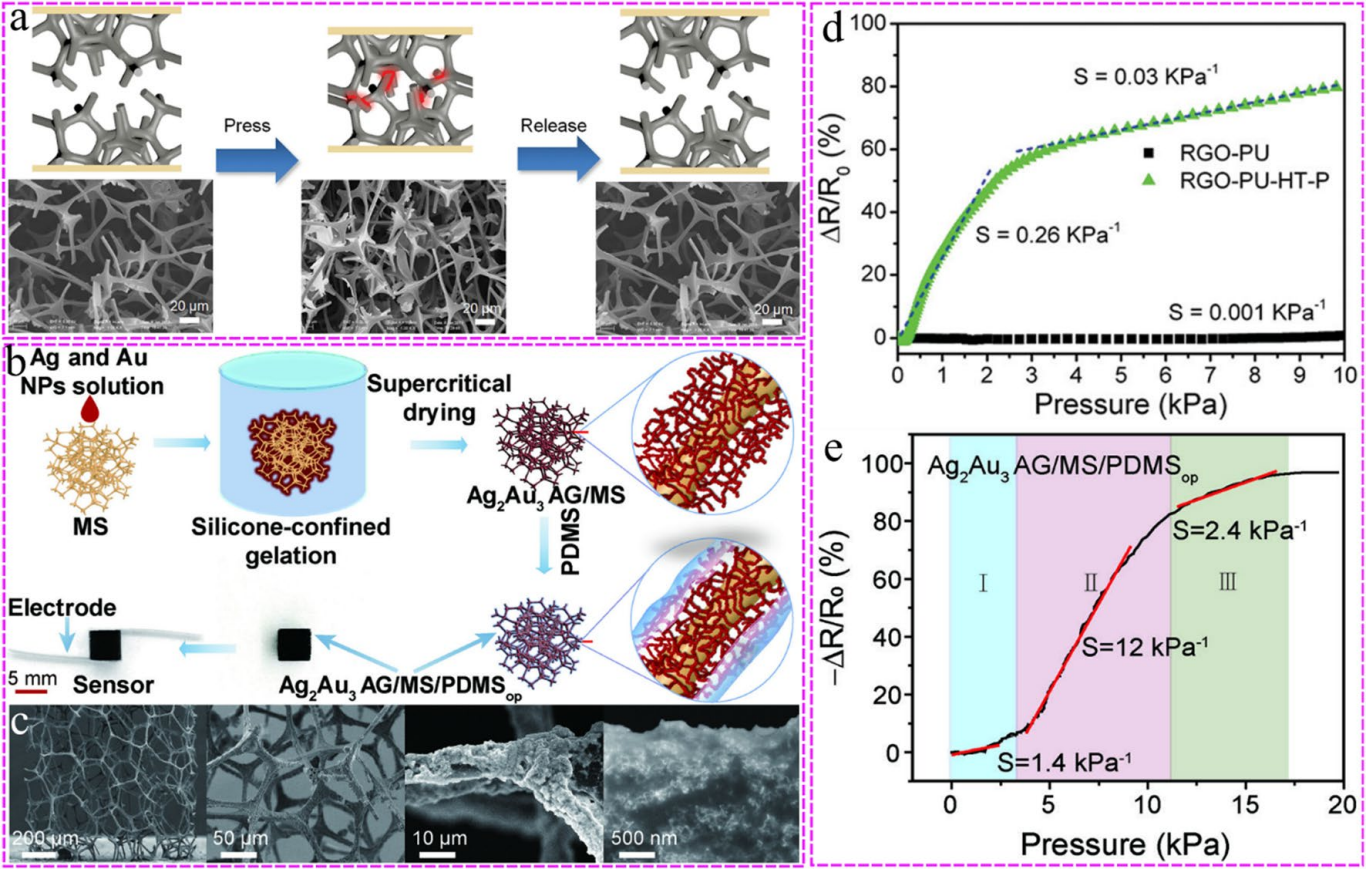
**a** The pressure-sensing model with fracture structure was studied under loading and releasing of pressure. SEM images shows the change of the contact area of the fiber network with the pressure. Reproduced with permission [116]. Copyright 2013, John Wiley and Sons. **b** Preparation flowchart of Ag_2_Au_3_ AG/MS/PDMS_op_ (aerogel/melamine sponge/polydimethylsiloxane) (‘op’ represents the optimized mass of PDMS (0.07 g)) flexible pressure sensor with hierarchical structure. Flexible pressure sensors can be made by installing copper foil electrodes on both sides of the Ag_2_Au_3_ AG/MS/PDMS_op_ sensing layer. Reproduced with permission [28]. Copyright 2022, John Wiley and Sons. **c** SEM images of Ag_2_Au_3_ AG/MS/PDMS_op_. Ag_2_Au_3_ AG/MS/PDMS_op_ takes sponge skeleton as the matrix, metal aerogel as the intermediate sensing layer, and PDMS as the protective layer to form a hierarchical structure. Reproduced with permission [28]. Copyright 2022, John Wiley and Sons. **d** The pressure response curves of reduced graphene oxide- polyurethane (RGO-PU) sponge and hydrothermally treated RGO-PU-HT-P sponge were compared. Obviously, the pressure sensitivity of hydrothermally treated RGO-PU sponge is much better than that of original RGO-PU sponge (Corresponds to Fig. **a**). Reproduced with permission [116]. Copyright 2013, John Wiley and Sons. **e** Analysis of theΔR/R_0_(%)-P (pressure) curve of Ag_2_Au_3_ AG/MS/PDMS_op_ flexible pressure sensor. And the sensitivity under different pressures was analyzed (Corresponds to Fig. **b**). Reproduced with permission [28]. Copyright 2022, John Wiley and Sons

Fracture effect is an obvious fracture phenomenon on sponge skeleton [117]. In order to further improve the contact change between conductive materials during compression and obtain a more sensitive sensor, researchers proposed the fracture effect based on the sponge framework [118]. Yang et al. [121] prepressed the polyurethane sponge, dipped graphene oxide solution under compression, released the load after reduction reaction, and the rGO conductive layer adhered to the PU sponge skeleton produced micro-wrinkles and micro-cracks. Utilizing the theory of contact mechanics and tunneling effect, the working mechanism of the sponge sensor was established, which effectively improved the performance of the sensor (S = 158.1 kPa^−1^). Another method is reflected in the fracture of 3D framework structures. High temperature hydrothermal treatment can be used to soften PU sponge, as some PU undergoes hydrolysis deformation. By compressing, a dense fracture structure can be obtained on the sponge skeleton. Compared with the original sponge sensor, the pressure sensitivity (S = 0.26 kPa^−1^) of the fractured sponge sensor increases by two orders of magnitude in the 0–2 kPa pressure range [116]. The preparation process and SEM images are shown in Fig. 5a, and the sensitivity analysis before and after fracture treatment are shown in Fig. 5d. In addition, there were a lot of imperfect frameworks in the conductive network constructed by adding brittle materials (such as cellulose nanocrystal) into the base blend solution, which could improve the sensitivity to pressure (GF = 1, in 80% compression strain range) [89].

#### Hierarchical structure of 3D framework structure

The hierarchical structure can be expressed as a 3D structure with a wide pore size distribution from small holes to large holes. It can be formed by adding templates with different pore structures or adding rough conductive materials on the basis of frameworks, forming a gradient structure [28, 59, 106, 108]. Small holes can provide higher surface structure, while large holes can cause large deformation of the sensing structure. The synergistic effect of the small holes and the large holes help to improve the resistance change under pressure applied. As shown in Fig. 5b, Li et al. [28] grafted Ag_2_Au_3_ metal aerogel onto the sponge skeleton with melamine sponge as the 3D framework and polydimethylsiloxane (PDMS) layer as the interface-reinforcing medium, developed a simple ‘interface locking strategy’ to form a 3D highly elastic and hierarchically gradient structure. There are pores of different sizes on the 3D framework, effectively improving the sensitivity and stability of the sensor under pressure. Preparation process and the SEM images of the hierarchical structure are shown in Fig. 5c, while Fig. 5e analyzes the sensitivity of the sensor with hierarchical structure under different pressures.

Table 2 summarizes the initial sensitivity, linear detection range and response time of 3D framework structure sensors prepared using different methods. From Table 2, it can be seen that the woodpile-shape pattern sensor prepared based on 3D printing technology has good performance, with a linear detection range of up to 175 kPa, even if its sensitivity is only 0.096 kPa^−1^. There are also flexible sensors designed with 3D spring and aerogel that only expand the linear detection range to 3 kPa with the sensitivity of 1.4 kPa^−1^. Nevertheless, compared to sensors designed with interface microstructures, their linear detection range has been significantly improved, but their sensitivity has also been significantly reduced. This is due to the relatively large pore of the 3D structure, which results in insignificant changes in electrical signals within the low-pressure range.

**Table 2:** Summary of pressure sensor parameters based on different 3D framework structure

Structure design	Sensitivity (KPa^−1^)	Linear sensing range (Pa)	Response time (ms)	References
Aerogel–hydrogel	1.1	140 K	17	[80]
MXene melamine sponge	9.97	15 K	180	[44]
Multilayered porous	50	4 K	200	[93]
Hierarchical sponge structure	5.93	5 K	15	[106]
Woodpile-shape	0.096	175 K	–	[58]
Hierarchical sponge	1.33	20 K	200	[108]
3D sponge	1	10 K	5	[109]
3D sponge + aerogel	1.4	3 K	85	[28]

With the improvement of 3D skeleton structure design methods, fracture effects and some hierarchical structure designs have significantly improved sensor sensitivity response under low pressure conditions. The hierarchical structure sensor designed by Lei et al. [106] increased sensitivity to 5.93 kPa^−1^ while maintaining a wide detection range of 5 kPa. These results indicate that the contradiction between sensitivity and detection range is gradually being resolved.

## Synergy of surface microstructure and 3D framework structure

**Fig. 6:**
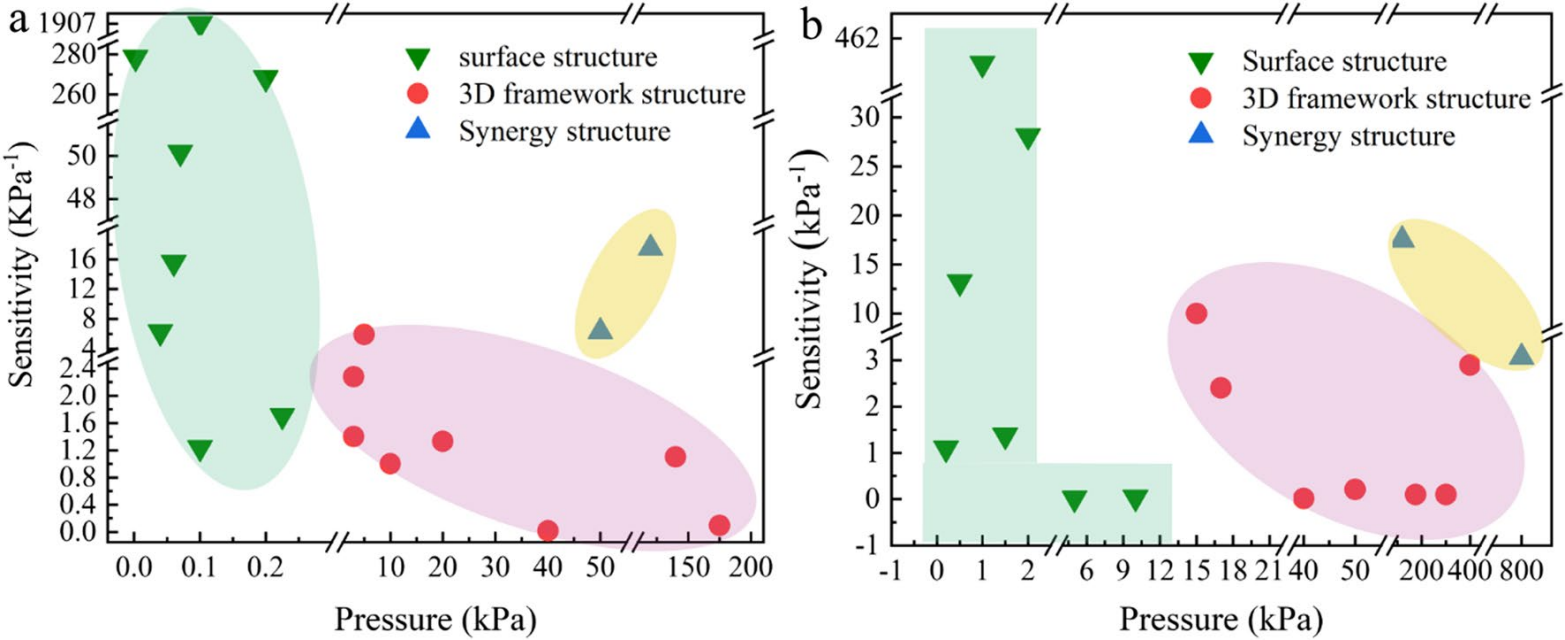
**a** Comparison of sensitivity and the pressure detection range of flexible pressure sensors designed based on interface microstructures (green) [51–53, 65, 70, 78, 120, 121], 3D framework structures (pink) [28, 44, 58, 80, 106–109] and synergy of interface and 3D structures in small pressure ranges (yellow) [50, 69]. **b** Comparison of sensitivity and the pressure detection range of flexible pressure sensors designed based on interface microstructure (green) [51–53, 65, 70, 78, 120], 3D framework structures (pink) [28, 44, 58, 80, 106–108] and synergy of interface and 3D structures over a large pressure range (yellow) [50, 69]

In summary, flexible sensors prepared with interface microstructures or 3D framework structures have their own advantages and disadvantages. As shown in Fig. 6a, flexible pressure sensors based on interface microstructures have relatively high sensitivity in the initial stage, but their linear detection range are within 0.3 kPa, which have certain limitations (as shown in the green background) [51–53, 65, 70, 78, 120, 121]. The flexible pressure sensors designed with 3D framework structure have relatively low sensitivity, basically less than 2.5 kPa^−1^, but their initial linear detection range have been significantly expanded (as shown in the pink background) [28, 44, 58, 80, 106–109]. Figure 6b shows the sensitivity analysis of the flexible sensor prepared with interface microstructure or 3D framework structure at the maximum detection range, and the results are similar to the initial stage of the sensor. The sensitivity analysis of flexible pressure sensors mainly designed with interface microstructures presents an ‘L’ shape, maintaining a relatively high sensitivity response under relatively small pressures, but there is a significant decrease compared to the initial stage. This result may be due to the saturation of microstructure deformation. The flexible pressure sensors mainly designed with 3D framework structure have a detection range of up to 400 kPa, but their sensitivities are still relatively low. In order to demonstrate the impact of different structures on the performance of sensors in more detail, Additional file 1: Table S1 compares the differences in sensitivity, linear detection range, response time, and maximum detection range of flexible sensors prepared by interface microstructure [51–53, 65, 70, 78, 120, 121], 3D framework structure [28, 44, 58, 80, 106–108, 122–125], and synergy of interface and 3D structures [50, 69]. From Fig. 6 and Additional file 1: Table S1, it can be seen that there is still a clear contradiction between the linear detection range and high sensitivity of flexible sensors prepared using interface microstructure design or three-dimensional frame structure design.

It is worth noting that the improvement of interface microstructure and three-dimensional framework structure effectively improves the contradiction between linear detection range and high sensitivity under certain conditions. On the basis of maintaining relatively high sensitivity, the initial linear detection range and effective detection range have been effectively expanded. In addition, in order to better adjust the relationship between sensitivity and detection range, researchers are also actively seeking other ways.

**Fig. 7:**
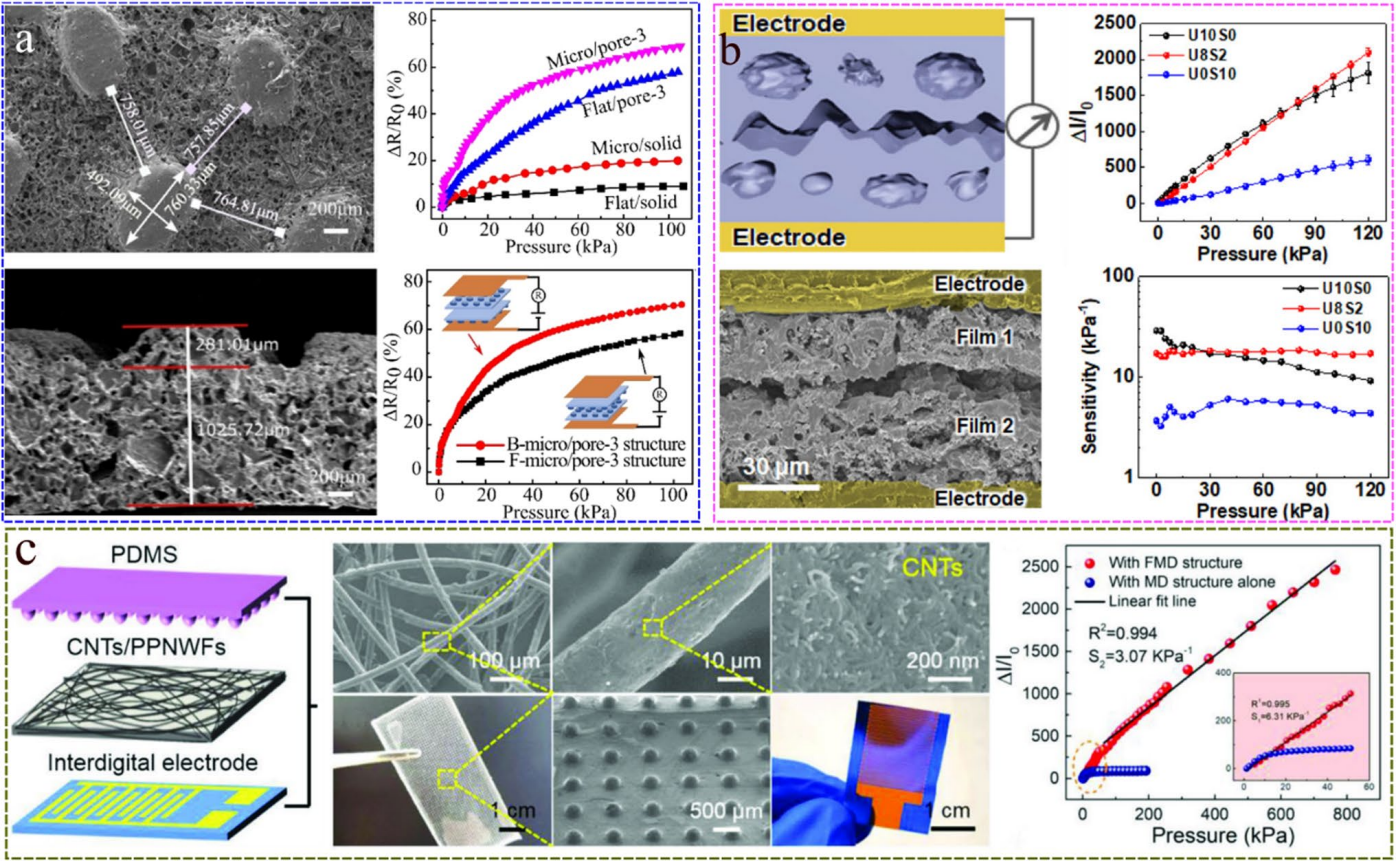
**a** SEM images of the top surface (upper left) and cross section (lower left) of the film with the micro/pore PDMS/MWCNTs structure. The pressure response curves of sensors with four different structures (flat/solid; micro/solid; flat/pore-3 and micro/pore-3; 3 represents the weight ratio of NaCl to PDMS prepolymer of 1 to 3) (upper right). The sensor with porous and surface microstructure has the most sensitive pressure response. The pressure response curves of different interlocking structures (with surface microstructure side interlocked face-to-face and with flat side stacked together) (lower right). The sensor with surface microstructure side interlocked face-to-face design has higher sensitivity. Reproduced with permission [38]. Copyright 2021, American Chemical Society. **b** Schematic diagram of the sensor based on the interlocked porous bilayer structure (upper left). The cross-section SEM image of the sensor based on the interlocked porous bilayer structure (lower left). Comparison of pressure response (upper right) and sensitivity (lower right) of pressure sensors with different particle content (U10S0, U8S2 and U0S10, where U and S represent the sea-urchin-shaped and spherical particles, respectively). Reproduced with permission [50]. Copyright 2020, American Chemical Society. **c** Schematic illustration of the structure with a nanofiber/interface micro-dome structured flexible pressure sensor (left). SEM images of nanofibers/CNTs with different magnifications (upper middle); Photographs and SEM image of the micro-dome structure at the interface of PDMS film and interdigital electrode (lower middle). Current variation curves and sensitivity analysis of sensors with FMD and MD structures under different pressures. Current variation curves and sensitivity analysis of fibrous and micro-dome (FMD) structured sensors and micro-dome (MD) structured sensors under different pressures (right). Reproduced with permission [69]. Copyright 2022, John Wiley and Sons

What can be achieved by combining the micro-structure design concept with the three-dimensional sponge structure? In order to solve this problem, our group has also done some work [38]. Through the synergistic effect of 3D porous structure and surface microstructure, a piezoresistive sensor with wide detection range and high sensitivity is designed. The porous structure was prepared by using sodium chloride particles as template, and the surface microstructure was obtained by using nonwovens as template. As shown in Fig. 7a, the synergistic effect of 3D porous structure and surface microstructure can effectively improve sensing performance. At the same time, the interlocking design further increases the sensitivity of the sensor to 10.805 kPa^−1^, and the detection range can reach 100 kPa. In addition, Kim et al. [50] designed an interlocking layer sensor with rough surface and internal microporous structure, and sprayed conductive nanoparticles with a sea-urchin-shaped spiky as electrodes. The combined structural formed by controllable structure from the nanoparticles, microscale porous structure and hierarchical surface morphology has obvious advantages in stress concentration and gradual deformation, and has achieved high sensitivity response in a wide pressure range (S = 17.5 kPa^−1^, 0.008–120 kPa), as shown in Fig. 7b. Cui et al. [69] effectively combined nanofibers with interface micro dome structures to form a synergistic effect of various deformation structures under pressure, making the sensor highly sensitive (6.31 kPa^−1^) and with a wide detection range (4.6–800 kPa), as shown in Fig. 7c. Under the synergistic effect of the structure, the sensitivity and linear detection range of the initial stage and maximum detection limit stage of the sensors are represented by the yellow background in Fig. 6. Its sensitivity and linear sensing range have been greatly improved, and it can maintain a high sensitivity response over a large detection range.

## Conclusion and prospect

In the past research, through a variety of structural design, the performance of the flexible piezoresistive sensor (especially the sensitivity) has been effectively improved. In this paper, the influence of the design of various structures on the sensitivity of the sensor were analyzed in detail, and the improvement scheme were proposed. These studies have played a significant role in promoting the development of sensors. Whether it is interface structure, 3D framework structure or collaborative design of different structures, it will affect the sensitivity and linear detection range of sensors. A balance between sensitivity and measurement range should be made based on specific application requirements. When conducting practical applications under low pressure conditions (physiological signals such as respiration and pulse), it is necessary to choose sensors with high sensitivity response to ensure the accuracy of experimental data. When performing human motion detection and other tasks with relatively high pressure, sensors with a wide detection range should be selected for analysis. For other performance indicators of sensors, repeatability, response time, etc. are important indicators. Therefore, the prerequisite for successful structural design is to ensure that the sensor has good cyclic stability. In sensors with microstructures, good cyclic stability was demonstrated, indicating that structural design has no significant impact on repeatability [61, 79, 90, 95].

However, there are still some problems in practical application. (1) The relationship between the linearity of sensitivity and the detection range. Although the design of some structures improves the sensitivity under certain conditions, the linear range is still small. (2) The adaptability of the sensor materials to the human body, including the reduction of sensor life caused by friction. (3) Influence of external environment on sensor performance (e.g., temperature, humidity, signal interference, etc.). (4) Real-time signal transmission. A perfect sensor needs a good signal receiving system, which requires interdisciplinary learning. For example, wireless design provides ideas for signal transmission, but needs further improvement.

There are still many issues that need to be further studied for flexible pressure sensors, and many fascinating structural and functional designs have not been developed. With the development of technology and the interdisciplinary development of materials science, chemistry, physics, and other fields, we will develop more reliable, efficient, and advanced multifunctional flexible sensors, opening up new opportunities in practical applications in medicine, electronics, optics, and other fields.

### Supplementary Information

**Additional file 1.** Table S1 compares the differences in sensitivity, linear detection range, response time, and maximum detection range of flexible piezoresistive sensors with different structural designs (interface microstructure, 3D framework structure and the synergy between interface and 3D framework structure). These results demonstrate that the design of the structure has a significant impact on sensor performance.

## Data Availability

All data generated or analyzed during this study are included in this published article [and its supplementary information files].
