# Supplementary material for: Research on high sensitivity piezoresistive sensor based on structural design
Source: Discov Nano. 2024 May 16;19(1):88. doi: 10.1186/s11671-024-03971-4 (PMC11098999; doi:10.1186/s11671-024-03971-4)
Supplement: Supplementary file 1 — Additional file 1. Table S1 compares the differences in sensitivity, linear detection range, response time, and maximum detection range of flexible piezoresistive sensors with different structural designs (interface microstructure, 3D framework structure and the synergy between interface and 3D framework structure). These results demonstrate that the design of the structure has a significant impact on sensor performance. [file 11671_2024_3971_MOESM1_ESM.docx]

**Supporting Information**

**Research on High Sensitivity Piezoresistive Sensor Based on Structural Design**

Wei Li,^#^ Xing Liu,^#^ Yifan Wang, Lu Peng, Xin Jin,* Zhaohui Jiang, Zengge Guo, Jie Chen, Wenyu Wang*

Table S1 compares the differences in sensitivity, linear detection range, response time, and maximum detection range of flexible piezoresistive sensors with different structural designs (interface microstructure, 3D framework structure and the synergy between interface and 3D framework structure). These results demonstrate that the design of the structure has a significant impact on sensor performance.

Table S1. Summary of Pressure Sensor Parameters Based on Different Structural Designs

|  | Structure Design | Sensitivity  (KPa^-1^) | Linear sensing range (Pa) | Minimum detectable range (Pa) | Maximum detection range (Pa) | Response time  (ms) | Ref |
| --- | --- | --- | --- | --- | --- | --- | --- |
| Interface  Micro-  structure | interlocking nanocone  arrays | 268.36 | 0-200 | 0.98 | 2000 | 56 | 51 |
|  | irregular microdomain | 50.17 | 0-70 | - | 1500 | 20 | 52 |
|  | conical structure | 1.1 | 1-60 | 12 | 200 | 300 | 54 |
|  | sharp micro-structure | 2000 | 100 | 0.075 | 1000 | 0.05 | 70 |
|  | micro-pyramid | 1.71 | 0-225 | - | 4500 | 6 | 65 |
|  | meso-dome arrays | 6.258 | 0-40 | - | 10300 | 20 | 78 |
|  | wrinkled structure | 278.5 | 22 | - | 500 | 20 | 120 |
|  | semi-spheroid-like protuberances | 1.24 | 150 | 1.3 | 7000 | - | 121 |
| 3D  Frame-  work  Structure | metal aerogel | 12 | 12000 | - | 17000 | 85 | 28 |
|  | sponge | 9.97 | 5000-15000 | - | 15000 | 180 | 44 |
|  | 3D printing | 0.096 | 0-175000 | - | 175000 | - | 58 |
|  | aerogel/hydrogel nanostructure | 1.1 | 120-400000 | 122 | 400000 | 17 | 80 |
|  | porous‑reinforcement  microstructure | 5.93 | 0-5000 | 0.0021g | 50000 | - | 106 |
|  | hierarchical structural  sponge | 0.29 | 0-2500 | - | 10000 | 54 | 107 |
|  | hierarchical gradient structure | 1.33 | 0-20000 | - | 100000 | 210 | 108 |
|  | melamine sponge | 48.26 | 12500-20000 | 20 | 20000 | 15 | 109 |
|  | cotton fabric | - | - | - | - | - | 122 |
|  | hydrogel | GF:6.67 | 0-1216% | - | 1216% | 120 | 123 |
|  | hydrogel | GF:3.93 | 1-600% | 1% | 600% | 500 | 124 |
|  | nanofiber film | GF:0.95 | 20% | - | 100% | - | 125 |
| Synergistically Micro-structured | Interlocked hierarchical micro/  nanostructures | 17.5 | 8-120000 | 8 | 120000 | - | 50 |
|  | fibrous and microdomed structure | 6.31 | 50000 | 4.6 | 800000 | 72 | 69 |

**Corresponding Authors**

**Xin Jin** - School of Materials Science and Engineering, Tiangong University, Tianjin 300387, China. Email: jinxin29@126.com

**Wenyu Wang** - School of Textile Science and Engineering, Tiangong University, Tianjin 300387, China. Email: wwy-322@126.com.

Authors

**Wei Li** - Lutai School of Textile and Apparel, Shandong University of Technology, Zibo 255000, China; Key Laboratory of Clean Dyeing and Finishing Technology of Zhejiang Province, Shaoxing University, Shaoxing, Zhejiang Province, People's Republic of China.

**Xing Liu** - School of Textile Science and Engineering, Tiangong University, Tianjin 300387, China.

Yifan Wang - School of Textile Science and Engineering, Tiangong University, Tianjin 300387, China.

**Lu Peng** - School of Textile Science and Engineering, Tiangong University, Tianjin 300387, China.

**Zhaohui Jiang** - Lutai School of Textile and Apparel, Shandong University of Technology, Zibo 255000, China; Key Laboratory of Clean Dyeing and Finishing Technology of Zhejiang Province, Shaoxing University, Shaoxing, Zhejiang Province, People's Republic of China; State Key Laboratory of Biobased Fiber Manufacturing Technology, China Textile Academy, Beijing, China.

**Zengge Guo** - Lutai School of Textile and Apparel, Shandong University of Technology, Zibo 255000, China.

**Jie Chen** - PLA Naval Medical Center, Shang Hai, China.

**Author Contributions**

W.L. and X.L. contributed equally. The manuscript was written through contributions of all authors. All authors have given approval to the final version of the manuscript.

**Notes**

The authors declare no competing financial interest.

**Acknowledgements**

Funding support from the Opening Project of Key Laboratory of Clean Dyeing and Finishing Technology of Zhejiang Province (Project Number: QJRZ2113), the Shandong Provincial Nature Science Foundation (grant no. ZR2020QE095), Open Fund of State Key Laboratory of Biobased Fiber Manufacturing Technology (No. SKL202204) and Project of the Naval Medical Center of China (Grand No.2023002) are acknowledged.
